# Single Image-Based Vignetting Correction for Improving the Consistency of Neural Activity Analysis in 2-Photon Functional Microscopy

**DOI:** 10.3389/fninf.2021.674439

**Published:** 2022-01-05

**Authors:** Dong Li, Guangyu Wang, René Werner, Hong Xie, Ji-Song Guan, Claus C. Hilgetag

**Affiliations:** ^1^Institute of Computational Neuroscience, University Medical Center Hamburg-Eppendorf, Hamburg, Germany; ^2^School of Life Sciences and Technology, ShanghaiTech University, Shanghai, China; ^3^Center for Biomedical Artificial Intelligence (bAIome), University Medical Center Hamburg-Eppendorf, Hamburg, Germany; ^4^Institute of Photonic Chips, University of Shanghai for Science and Technology, Shanghai, China; ^5^School of Health Science and Engineering, University of Shanghai for Science and Technology, Shanghai, China; ^6^Institute of Psychology, Chinese Academy of Sciences, Beijing, China; ^7^Department of Health Sciences, Boston University, Boston, MA, United States

**Keywords:** vignetting correction, functional microscopic imaging, neural activity, image analysis, imaging artifacts

## Abstract

High-resolution functional 2-photon microscopy of neural activity is a cornerstone technique in current neuroscience, enabling, for instance, the image-based analysis of relations of the organization of local neuron populations and their temporal neural activity patterns. Interpreting local image intensity as a direct quantitative measure of neural activity presumes, however, a consistent within- and across-image relationship between the image intensity and neural activity, which may be subject to interference by illumination artifacts. In particular, the so-called vignetting artifact—the decrease of image intensity toward the edges of an image—is, at the moment, widely neglected in the context of functional microscopy analyses of neural activity, but potentially introduces a substantial center-periphery bias of derived functional measures. In the present report, we propose a straightforward protocol for single image-based vignetting correction. Using immediate-early gene-based 2-photon microscopic neural image data of the mouse brain, we show the necessity of correcting both image brightness and contrast to improve within- and across-image intensity consistency and demonstrate the plausibility of the resulting functional data.

## Introduction

Modern microscopic imaging techniques provide unique insights into the structure and functioning of complex biological neural systems. For many applications—ranging from whole-brain imaging (Gao et al., 2019) to single-cell isolation (Brasko et al., 2018)—it has been shown that illumination correction, that is, the removal of uneven illumination of a scene and specimen, facilitates image interpretation, improves visibility particularly of fine structures and is a pivotal pre-processing step for subsequent image analysis (Smith et al., 2015). Illumination correction is, therefore, often an essential component of microscopy imaging setups (Model, 2014; Khaw et al., 2018) and image post-processing (Ljosa and Carpenter, 2009; Caicedo et al., 2017; Todorov and Saeys, 2019).

A common and major illumination artifact in microscopic images is the *vignetting artifact*, which reflects a (usually radial) decrease of the image brightness, its contrast, or the saturation from the image center toward the periphery (Leong et al., 2003). Correction approaches can be divided into prospective methods that exploit reference images (Young, 2000), retrospective multiple image-based correction (Singh et al., 2014; Smith et al., 2015; Peng et al., 2017) and single image-based strategies. Reference and multiple image-based methods are considered most reliable (Smith et al., 2015), but are not always applicable due to application-specific constraints (e.g., data availability). At this point, single image correction strategies come into play (Leong et al., 2003; Zheng et al., 2008).

In the present report, we focus on high-resolution functional microscopic imaging and its application to studying the functioning of biological neural systems. Techniques such as calcium indicator-based (O’Donovan et al., 1993; Svoboda et al., 1997; Wachowiak and Cohen, 2001; Stosiek et al., 2003; Birkner et al., 2017; Tischbirek et al., 2019; Yildirim et al., 2019) or immediate early gene (IEG)-based imaging (Barth et al., 2004; Wang et al., 2006, 2019; Barth, 2007; Xie et al., 2014; Franceschini et al., 2020) allow, in principle, an interpretation of the read-out of local image intensity as neural activity. While, intuitively, a quantitative comparison of neural activity of, for example, local neuron populations is susceptible to illumination artifacts, illumination correction has, so far, been widely neglected or unreported in respective studies. A potential reason might be that established reference- and multiple image-based correction methods result in the same correction effect at any given location on different microscopic images. This in turn may compromise the analysis of interrelations of neural activity patterns by inducing artificial correlations between the modified images.

To overcome this issue,

• we develop and propose a straightforward single image-based vignetting correction protocol and
• demonstrate the value of the vignetting correction using single channel IEG microscopic images of the mouse brain.

Due to the application-inherent lack of ground truth data to evaluate the effect of the illumination correction, we focus on data plausibility: In the presence of a vignetting artifact, the functional data contain a significant center-periphery bias of neural activity measures that, for the performed experiments, cannot be accounted for by the underlying biology. Considering the reduction of the center-periphery bias in neural activity as well as consistency of absolute and relative activity changes over time as proxies of success, we demonstrate that the vignetting correction substantially improves data plausibility. Going beyond the standard approach of only taking into account the brightness of the image background (Leong et al., 2003), we further show that the largest improvement in data plausibility is achieved by correction of both image brightness and contrast.

## Materials and Methods

### Imaging and Image Data

The present study is based on single channel 2-photon imaging mouse data as detailed in Xie et al. (2014). The mouse strain was BAC-EGR-1-EGFP [Tg(Egr1-EGFP)GO90Gsat/Mmucd from the Gensat project, distributed by Jackson Laboratories]. Animal care was in accordance with the institutional guidelines of and the experimental protocol approved by Tsinghua University. To allow for *in vivo* imaging, a cranial window was implanted between the ears of 3–5 months old mice. Data recording started a month later. EGFP fluorescent intensity was imaged using an Olympus Fluoview 1200MPE with pre-chirp optics and a fast AOM mounted on an Olympus BX61WI upright microscope, coupled with a 2 mm working distance, 25 × water immersion lens (numerical aperture 1.05). The animals were anesthetized 1 h after they explored a multisensory environment. Under the given experimental conditions, anesthetization is known to have very little effect on protein expression (Bunting et al., 2016), and protein expression to reflect neural activity (Xie et al., 2014). We randomly selected one image stack with measurements acquired at 2 days (interval in between: 5 days) to showcase our analysis. The stack belonged to the primary visual (VISp) area. Stack size was 512 × 512 pixels with a pixel edge length of 0.996 μm for the in-plane slices, containing 350 slices in z-direction with a spacing of 2 μm. Neuron segmentation was performed as described in Xie et al. (2014). The segmentation was performed in the uncorrected image data, to avoid neuron position mismatches between the unprocessed and the corrected image data. The neuron center positions were used to evaluate neural activity.

### Illumination Correction Pipeline

**FIGURE 1:**
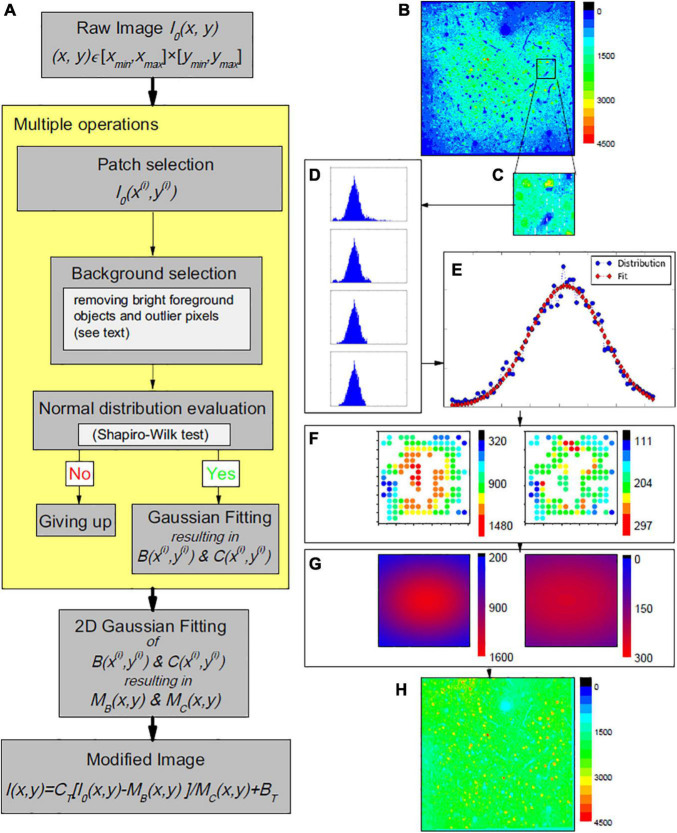
**(A)** Working pipeline. **(B–H)** One example of the proposed vignetting correction of a 2-photon microscopy image of mouse area VISp at layers II/III depth (200 μm depth). **(B)** The original image, i.e., before correction. **(C)** Example patch selection. **(D)** Histograms of image pixel intensities, illustrating the effect of the performed outlier reduction. In top-down direction: first, histograms of all pixels in the patch; second, after dropping off the long tail; third, after dropping off some data of certain values at both ends; last, after dropping off additional data of certain amounts at both ends (details given in the source code). At the end, the similarity to a normal distribution estimated by a Shapiro-Wilk test is 0.996, larger than the threshold we used (0.98). **(E)** Corresponding Gaussian fitting of the background intensity for the patch pixels. **(F)** Supporting point (i.e., the patch centers) brightness *B*(*x*^(*i*)^,*y*^(*i*)^) (left panel) and contrast *C*(*x*^(*i*)^,*y*^(*i*)^) (right panel). **(G)** The estimated global brightness distributions *M*_*B*_(*x*,*y*) and *M*_*C*_(*x*,*y*) (left and right panel). **(H)** The final modified image.


Our proposed single image-based pipeline for vignetting correction in 2-photon functional microscopy data is outlined in Figure 1A. Let a raw, i.e., measured and potentially post-processed, single channel microscopic image (an example is shown in Figure 1B) be denoted by *I*_0_(*x*,*y*) with (*x*,*y*) ∈ [*x*_*min*_,*x*_*max*_]×[*y*_*min*_,*y*_*max*_] = :Ω⊂ℝ^2^. In the following, we approximate the relationship between *I*_*0*_ and the sought true image and neural activity *I*, respectively, by

(1) $$I\,\left(x,y\right)=C_{T}\,\frac{I_{0}\,\left(x,y\right)-M_{B}\,\left(x,y\right)}{M_{C}\,\left(x,y\right)}+B_{T}$$

with *M*_*C*_(*x*,*y*) and *M*_*B*_(*x*,*y*) as a spatially varying gain or contrast distribution and a spatially varying background brightness, respectively, that are to be estimated and compensated during the illumination correction. *B*_*T*_ and *C*_*T*_ are pre-defined additive and multiplicative constants to bring the corrected image into a desired value range (Piccinini et al., 2012), and are not part of the correction process. With *C*_*T*_ = 1 and *B*_*T*_ = 0, Equation 1 corresponds to the standard notation as, for instance, used by Smith et al. (2015).

The proposed correction approach follows the common assumption that vignetting effects can be approximated by Gaussian functions. Leong et al. (2003) for instance, described the uneven illumination as an additive low frequency signal and approximated it by an isotropic Gaussian distribution with large standard deviation. The distribution parameters were, however, chosen *ad hoc* and image-specific (Leong et al., 2003). Here, we also model *M*_*B*_(*x*,*y*) and *M*_*C*_(*x*,*y*) by 2-dimensional (2D) Gaussian functions, but estimate the distribution parameters by analysis of *I*_0_(*x*,*y*).

To be able to efficiently cope with potentially large image data sets, we employ a patched-based approach, consisting of two main steps:

[STEP 1] patch-based robust estimation of local background brightness and contrast for a sufficient amount of supporting points distributed across *I*_0_(*x*,*y*), and

[STEP 2] estimation of *M*_*B*_(*x*,*y*) and *M*_*C*_(*x*,*y*) based on the supporting point brightness and contrast values.

#### STEP 1: Patch-Based Estimation of Local Brightness and Contrast

In line with most existing methods in the given context, we focus on the image background to estimate the vignetting functions (Leong et al., 2003; Charles et al., 2008; Chalfoun et al., 2015). Let $I_{0}^{\left(i\right)}$ denote a patch of *I*_*0*_ with a patch center (*x*^(*i*)^,*y*^(*i*)^) ∈ Ω and a domain [*x*_*i*_−λ_*x*_/2,*x*_*i*_ + λ_*x*_/2]×[*y*_*i*_−λ_*y*_/2,*y*_*i*_ + λ_*y*_/2] = :Ω_i_⊂Ω, with λ_*x*_ and λ_*y*_ as the side lengths of the patches (Figure 1C). Subsequently, we assume an ordered sequence of patches ${\left(I_{0}^{\left(i\right)}\right)}_{i=1,\ldots ,n}$ to be given that covers *I*_*0*_. However, the proposed approach is applicable to any patch sampling strategy. The patch centers are considered the supporting points for [STEP 2]; the associated brightness and contrast values were computed based on a histogram analysis of the intensity values of the patch pixels. Starting with the original histogram for a patch $I_{0}^{\left(i\right)}$, contributions by high intensity, that is, foreground objects such as highly active neurons that lead to a long-tailed intensity distribution, were removed following the approach of Clauset et al. (2009) and Alstott et al. (2014) that seeks to find the minimum histogram data point to optimally fit a power law to the right tail of pixel intensity distribution. The histogram data points above this intensity value were discarded from further analysis. Further remaining high and low intensity values were removed by focusing on the central parts of the histogram and the intensity distribution, respectively. The intensity distribution of the remaining data points was tested for normality using the Shapiro-Wilk test (Shapiro and Wilk, 1965); only patches with a resulting value above a pre-defined threshold of the test statistic were considered valid supporting points for [STEP 2] (Figure 1D). The corresponding local brightness and contrast values *B*(*x*^(*i*)^,*y*^(*i*)^) and *C*(*x*^(*i*)^,*y*^(*i*)^) were approximated by the expectation and the standard deviation of the fitted normal distribution (Figure 1E). For further details and parameter values used in our study, we refer to the source code (see Data Availability).

#### STEP 2: Approximation of *M*_*B*_(*x*,*y*) and *M*_*C*_(*x*,*y*)

Based on the patches $I_{0}^{\left(i\right)}$, *i* ∈ *I*⊆{1,…,*n*}, for which the intensity distributions passed the Shapiro-Wilk test, the corresponding set of patch estimates *B*(*x*^(*i*)^,*y*^(*i*)^) and *C*(*x*^(*i*)^,*y*^(*i*)^) (Figure 1F) were used to fit 2D-Gaussian distributions to estimate the sought distributions *M*_*B*_(*x*,*y*) and *M*_*C*_(*x*,*y*)(Figure 1G).

### Experiments and Evaluation

**FIGURE 2:**
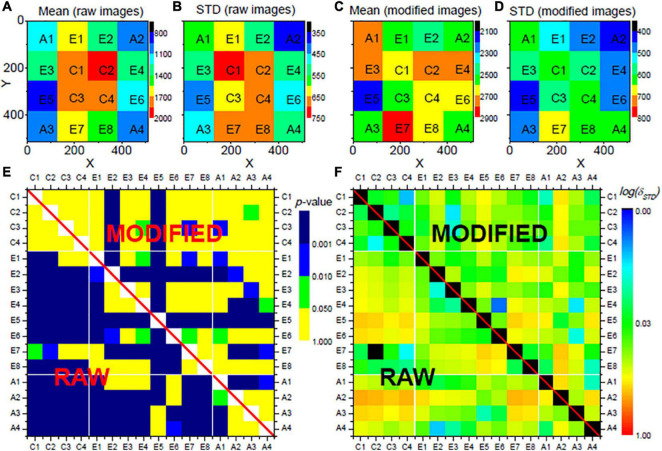
Evaluation results for the proposed vignetting correction for the neural activities *X*_*l*_ in layers II/III of a mouse VISp area. The mouse was located in the home cage. The images stack was evenly divided into 16 regions on x-y plane, as shown in **(A–D)**. **(A)** Shows the mean activities of all neurons within each region sampled from the raw images, **(B)** shows their standard deviations. **(C,D)** Show the mean values and the standard deviations sampled from the modified images. **(E)** Shows the comparison of the neural activities between each pair of regions, where colors stand for the *p*-values of a respective *t*-test with Bonferroni correction. **(F)** Shows the comparison of the distributions of the neural activities between each pair of regions, where colors indicate δSTD with logarithm scale. In **(E,F)**, the lower rectangles, i.e., the values below the diagonal, show the comparison with the data from the original images. The upper triangles represent corresponding data from the modified images. All parameters for the vignetting correction are the same as applied in Figure 1.

To evaluate plausibility of the image correction, the slices of the image stack (see section “Imaging and Image Data”) were evenly divided into 4 × 4 = 16 regions (see Figures 2A–D): four center regions (C1 to C4), eight edge regions (E1 to E8), and four angle regions (A1 to A4). If a vignetting effect exists, the comparison of neuronal activity derived for the different regions shows a significant bias, by which the center regions have high activity, which decreases radially (i.e., highest activity differences between the C and the A regions). Correspondingly, after successful correction of the vignetting effect, the bias should be significantly reduced. The slices were further grouped into four laminar compartments: layer II/III slices (95 slices in our selected stack), layer IV slices (41 slices), layer V slices (94 slices), and layer VI slices (97 slices). The layers were assigned manually.

Motivated by functional neuroimaging studies (O’Donovan et al., 1993; Svoboda et al., 1997; Wachowiak and Cohen, 2001; Stosiek et al., 2003; Barth et al., 2004; Wang et al., 2006, 2019; Barth, 2007; Xie et al., 2014; Bunting et al., 2016; Birkner et al., 2017; Moeyaert et al., 2018; Tischbirek et al., 2019; Yildirim et al., 2019), we considered the following measures to analyze the impact of the vignetting effect and the reduction thereof:

• **Neural activity:** Based on the segmentation of the neurons (see Sec. “Imaging and Image Data”), the activity *X*_*l*_ of the *l-*th detected neuron (*l* = 1,…,*n*_*l*_) is computed as the intensity of the center pixel of the segmentation mask.
• **Absolute changes of neural activity over time:** Neural activity measurements were available for two different days, denoted as *day1* and *day0*, with an intervening interval of 5 days. In addition to the neural activity, we also evaluated the absolute activity changes Δ*X*_*l*_ = *X*_*l*_(*day*1)−*X*_*l*_(*day*0).
• **Relative changes of neural activity:** In addition, we evaluated the relative neural activity changes δ*X*_*l*_ = [*X*_*l*_(*day*1)−*X*_*l*_(*day*0)]/[*X*_*l*_(*day*1) + *X*_*l*_(*day*0)].

The three measures *X*_*l*_, Δ*X*_*l*_ and δ*X*_*l*_ were separately evaluated for the 16 regions (C1-C4, E1-E8, A1-A4) and the four laminar compartments (II/III, IV, V, VI) before and after vignetting correction. Results are given as mean and standard deviation (STD) of the measures for the individual regions and laminar compartments. Significant differences between the measures of two different regions were evaluated separately for the different laminar compartments, applying *t*-tests with Bonferroni correction of the *p*-values. Moreover, for each pair of regions, the relative difference δ_*STD*_ of the standard deviations of the considered measure values within the individual regions was computed for the different laminar compartments. Thus, for each laminar compartment, in total *n*_*c*_ = 6 center-center (C-C) comparisons, *n*_*c*_ = 28 edge-edge (E-E) comparisons, *n*_*c*_ = 6 angle-angle (A-A) comparisons, *n*_*c*_ = 32 center-edge (C-E) comparisons, *n*_*c*_ = 32 edge-angle (E-A) comparisons, and *n*_*c*_ = 16 center-angle (C-A) comparisons were performed.

To obtain further insights into a potential vignetting-associated center-to-periphery bias before image correction, we also evaluated the fraction of region pairs with non-significant differences (*p*≥0.01 after Bonferroni correction) for the different measures on a region-type level (i.e., focusing on all C-C, E-E, A-A, C-A, C-E, etc. comparisons). Let *c*_*p*_ = *n*_*c*(*p*≥0.01)_/*n*_*c*_ denote the fraction of the total number of region type-specific comparisons (e.g., *n*_*c*_ = 16 for C-A comparisons) and the number *n*_*c(p≥0.01)*_ of corresponding non-significant comparisons. Then, the ratio Δ*C* = *c*_*p*_/⟨*c_p_*⟩*_same_* was computed, with ⟨*c_p_*⟩*_same_* as the average value of *c*_*p*_ values of the C-C, E-E, and A-A comparisons. The hypothesis was that, in the presence of a vignetting effect, Δ*C* values for, for example, the C-E and C-A comparisons are considerably smaller than one; after successful correction, the values should become closer to one. In particular, we assumed the comparison of Δ*C* values for C-A and E-E comparisons before and after correction to indicate presence and successful correction of the vignetting effect.

Similar to Δ*C*, we also evaluated differences of the relative standard deviations δ_*STD*_ on a region-type level. With $\bar{\delta_{S\,T\,D}}$ as average δ_*STD*_ value for all pairs of two of the region types C, E or A for a specific layer compartment, we computed $\Delta \,S\,T\,D=\bar{\delta_{S\,T\,D}}/{\left\langle \bar{\delta_{S\,T\,D}}\right\rangle }_{s\,a\,m\,e}$, with ${\left\langle \bar{\delta_{S\,T\,D}}\right\rangle }_{s\,a\,m\,e}$ denoting the average value of $\bar{\delta_{S\,T\,D}}$ for C-C, E-E, and A-A comparisons. A correction of a vignetting effect would, for instance, lead to significant reduction of Δ*STD* for C-A comparisons, especially when compared to Δ*STD* for E-E comparisons.

All measures were evaluated for the original image data as well as after vignetting correction according to Equation 1. In addition, we also applied a brightness-only correction, using


(2) $$I\,\left(x,y\right)=I_{0}\,\left(x,y\right)-M_{B}\,\left(x,y\right)+B_{T}$$

as well as a contrast-only correction,


(3) $$I\,\left(x,y\right)=C_{T}\,\frac{I_{0}\,\left(x,y\right)-M_{B}\,\left(x,y\right)}{M_{C}\,\left(x,y\right)}+M_{B}\,\left(x,y\right),$$

to illustrate the respective effects.

## Results

Subsequent results are based on automatically segmented 14.662 neurons. Within each region of the laminar compartments, on average 229 (STD: 78; range: 50–376) neurons were segmented. An example of an image slice before and after vignetting correction is shown in Figures 1B,H, respectively. In the original data, a strong image intensity decrease from the center toward the periphery can be seen. This gradient, in turn, leads to a larger number of significant differences of the measures derived for the different regions and region-types, indicated by blue and green fields in the lower triangles of Figure 2E (focusing on neural activity *X*_*l*_ itself), Supplementary Figure 1 (absolute changes Δ*X*_*l*_of the neural activity), and Supplementary Figure 2 (relative changes δ*X*_*l*_ of neural activity), respectively, for the layers II/III of the considered VISp area. Corresponding representations for layers IV, V, and VI are shown in Supplementary Figures 3–17. Analogously, large δ_*STD*_ values are present for the original data (see lower triangle of Figure 1F and Supplementary Figures 1–17). In particular, the C-A comparisons exhibit a larger number of significant differences and larger δ_*STD*_ values than the “within-type” comparisons, that is, the C-C, E-E, and A-A comparisons. Corresponding C-E and E-A values are somewhere in between. This finding clearly indicates the existence of vignetting effects in the original data.

After applying the proposed image correction procedure, the abovementioned effects are substantially diminished: The number of significant region-to-region differences of the evaluated measures is considerably reduced; similarly, the δ_*STD*_ values are more consistent between the regions of different types (e.g., C-A and E-A; see upper triangle in Figures 1E,F and Supplementary Figures 1–11). This effect, however, is much less pronounced if only an intensity or only a contrast correction is applied (Supplementary Figures 12–17).

**FIGURE 3:**
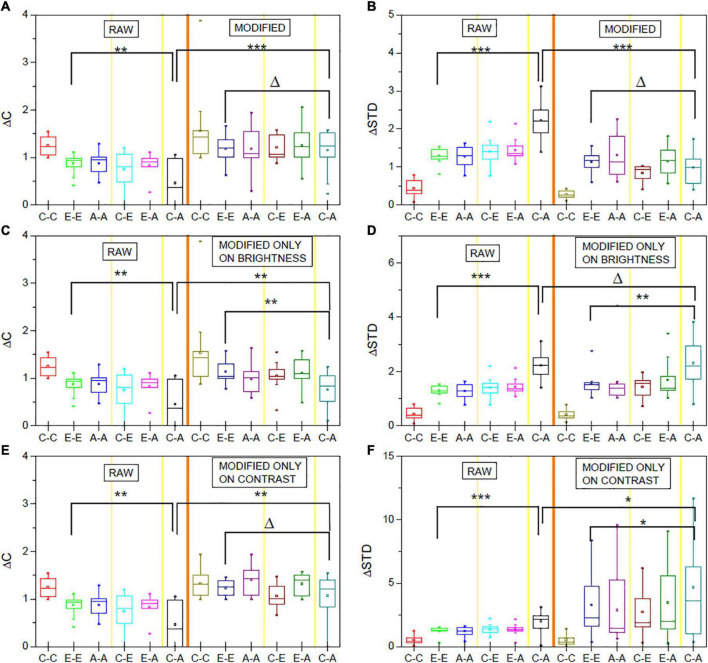
ΔC and ΔSTD values for the original (RAW) and corresponding values after vignetting correction. **(A,B)** Represent the effect if performing brightness and contrast correction. In **(C,D)** (right sides), only brightness was corrected. Similarly, **(E,F)** show the data for contrast-only correction. The shown data are averaged over the obtained normalized data for the three studied measures (neural activity, absolute differences of neural activity, relative differences) and the different layer compartments. ^Δ^*p* > 0.05, *p < 0.05; **p < 0.01, ***p < 0.001, *t*-test.

In line with these observations and the hypothesis described in section “Experiments and Evaluation,” before the vignetting effect correction, the Δ*C* values for the C-A comparisons are significantly lower (*p* < 0.01) and the Δ*STD* values significantly higher than corresponding values for E-E comparisons (*p* < 0.001). After image correction, the differences are no longer significant, associated with a significant increase of Δ*C* and decrease of Δ*STD* (both *p* < 0.001), due to the C-A values after image modification (Figures 3A,B).

Figures 3C–F further reveal (similar to Supplementary Figures 12–17) that brightness-only or contrast-only correction does not sufficiently correct for the vignetting effect. If only a brightness correction is applied, Δ*C* values are, for instance, still significantly lower for C-A comparisons than for E-E comparisons. If only a contrast correction is applied, the C-A Δ*STD* values are still significantly higher compared to the E-E values (*p* < 0.05).

## Discussion

### Center-Periphery Bias and Data Rationality

Correction of uneven illumination, and in particular a correction of the vignetting effect, has become a standard procedure in *structural* neural microscopy image processing (Brasko et al., 2018; Gao et al., 2019). In contrast, the application of such corrections to corresponding *functional* neural microscopy images is rarely reported, and the influence and effects of respective procedures remain unclear so far. This lack of studies may, in part, be due to a lack of ground truth data that could be used for evaluation purposes. However, we hypothesize that the intrinsic consistency and plausibility of the data is an appropriate indicator for the existence and the success of a correction of vignetting effects. While neural microscopy data and commonly derived measures, such as neural activity and absolute or relative activity, change over time and certainly exhibit spatially varying distributions, there is no convincing physiological explanation for the existence of a distribution of the measures with a center-periphery bias—that is, a vignetting effect. A reduction, and ideally the elimination, of a center-periphery bias can, therefore, be studied to assess the success of a correction in functional microscopic neural images.

In this work, we implemented a straightforward single image-based correction method, and used IEG 2-photon microscopic neural image data of the mouse brain to demonstrate that such data are affected by vignetting effects (see Figure 1B), and that the proposed method corrects for the resulting center-periphery bias of the image intensity and derived measures (see, e.g., Figure 2). We further illustrated that the most effective correction of the vignetting effect requires correction of both the brightness and the contrast of the image background. The corresponding code is publicly available to allow re-use of the proposed solutions (see section “Data Availability Statement”).

### Effect of Patch Size

The patch size is a key parameter of the proposed vignetting correction protocol. The individual patches should contain a sufficient number of background pixels to allow for histogram fitting of sufficient quality (as evaluated by the Shapiro-Wilk test). The patches should, however, be sufficiently small and compared to the entire slice to guarantee existence of appropriate sampling points for the subsequent 2D Gaussian fitting and estimation of *M*_*B*_(*x*,*y*) and *M*_*C*_(*x*,*y*), respectively.

**FIGURE 4:**
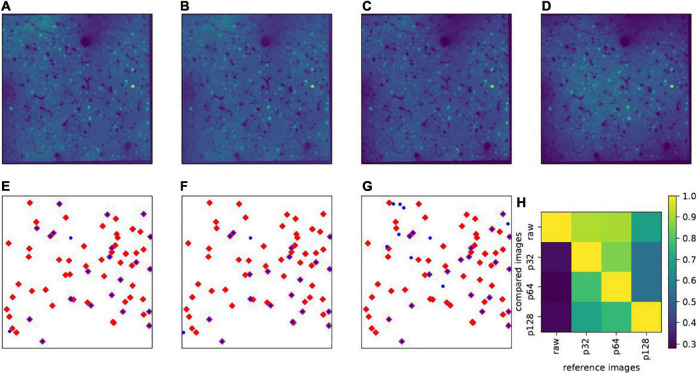
Effect of patch size. Upper panels show the same example slice (in layers II/III) with different patch sizes for vignetting correction: **(A)** Patch size 32×32 pixels (denoted as p32); (B) 64×64 pixels (p64); **(C)** 128×128 pixels (p128); and **(D)** raw images without vignetting correction. In **(E–G)**, the blue dots show the projections of all highly active cells (δ*X*_*l*_ is larger than the mean value plus three times the standard deviation) in layers II/III on the x-y plane, calculated based on p32, p64, and p128, respectively, and the red diamonds represent the highly active cells calculated based on the raw images. **(H)** Shows the overlap rates (|H*_i_*∩H*_j_*|/|H*_i_*|) of highly active cells between any pair of the aforementioned images, where H*_i_* denotes the set of highly active cells from the reference image, H*_j_* from the image used for comparison, and |⋅| denotes the cardinality of the set.

Thus, the selection of the patch size is a trade-off and depending on the size of the entire slice. Correction results with different patch sizes are illustrated in Figure 4 (Figure 4A: patch size 32×32 pixels; Figure 4B, 64×64pixels; Figure 4C, 128×128 pixels; and Figure 4D: raw image). Figures 4E–G illustrate the influence of the patch size selection on the data analysis. Throughout this work, including the example in Figure 4, the Shapiro-Wilk test threshold was always set to 0.98, and the slice size was always 512×512 pixels. For these parameters, a patch size of 32×32 pixels appeared visually sufficient for the correction process and the vignetting effect significantly reduced (see Figures 4A,D). A smaller patch size of 16×16 pixels usually led to failure of the Shapiro-Wilk test. A patch size of 64×64 pixels (Figure 4B) yielded very similar results to 32×32 pixel patches. However, for patch sizes of 128×128 pixels and larger, a residual vignetting effect is visible (Figure 4C).

These considerations may be particularly relevant in the context of identifying highly active cells, which are used for the definition of so-called memory traces (Xie et al., 2014). Examples of such highly active cells (δ*X*_*l*_ larger than the mean activity values plus three times the standard deviation) are given in the lower panels of Figure 4. From those panels, it is easy to note that, even with different, but proper patch sizes, the highly active cells have very high overlaps (32×32*pixelsversus* 64×64*pixels*). In addition, we see in this example that the highly active cells calculated from the corrected images with proper patch sizes roughly constitute a subset of those apparent in raw images, where the ones calculated from raw images include many false positive cases (Figure 4H). This phenomenon may not always be present, as it is sensitive to the mathematical definition of how the highly active target cells are calculated, but it is conspicuous enough to suggest the existence of false positive detections in uncorrected images.

### Multiple Image- vs. Single Image-Based Correction

As described in the Introduction, vignetting correction approaches can be divided into two main groups: multiple image-based vs. single image-based correction. A prerequisite of multiple image-based vignetting correction is, obviously, the availability of multiple images. Multiple image correction is often considered more reliable than single image correction, but also comes with some potential disadvantages. One aspect is that multiple image-based correction typically exploits the intensity distribution of pixels at the same spatial position but in different images. This relationship, in turn, implicitly assumes and induces a (potentially artificial) correlation between the images that are processed. The artificial correlation may not be of utmost relevance when processing structural microscopy data. In functional microscopy neural data analysis, however, existence or absence of a correlation of signals from different images is often at the core of the given research question (Xie et al., 2014; Wang et al., 2019). Thus, it has to be ensured that the observed correlations are of a biological nature, rather than artificial.

This requirement, in turn, renders single image-based image correction particularly useful for functional microscopic neural data analysis; it is applicable even if the exact form of the vignetting effects varies day-by-day. For functional neural microscopy imaging, such day-by-day variations exist due to several reasons. For instance, one such reason is that blood vessel volume and throughput can vary, with a substantial impact on light intensity (Shih et al., 2012). It is, in principle, technically possible to modify the craniofacial light intensities to a certain degree by adjusting the laser parameters, but this adjustment only eliminates the effects caused by vessels over the brain surface. Since there are far more unpredictable components influencing the light intensities inside the brain, for instance., the irregular distribution of blood vessels and dynamic locations of glia or immune cells, it is hardly possible to pre-set proper parameters at different depths to unify the intensities across days for both the superficial and deeper slices at the same time. In addition, daily shifts of the position of the mouse brain in the microscope induce additional sources of error. The errors are corrected by slightly rotating the images after scanning, but this inevitably also changes the appearance of the vignetting effects. Moreover, the optical pathways of the laser cannot be guaranteed to be sufficiently stable on long time scales. If the experiments last too long, this may also be a reason for daily variations of observed vignetting effects.

Furthermore, from a methodical perspective, underlying mechanisms of multiple and single image-based correction approaches are often, to a certain degree, similar. For example, Smith et al. (2015) simultaneously modified image brightness and contrast, similar to our proposed approach. For both approaches, the image modification is based on the analysis and transformation of a pixel intensity distribution—with the difference that Smith et al. (2015) determined the distribution of pixels from different images, but at the same spatial position. In contrast, we focus on patch pixels of a single image; in addition, we exclude bright objects (here: mainly pixels of high activity) to ensure an unbiased estimation of the background intensity distributions.

Thus, for single image-based vignetting correction, the multiple image-based prior information is essentially replaced by the hypothesis that the image background intensity and the image contrast distribution can be approximated by two-dimensional Gaussian distributions. The two-dimensional Gaussian fitting to the image background for single image-based vignetting correction was already proposed by Leong et al. (2003). In the present work, their idea was extended by additional consideration of the image background contrast. The described results indicated that both brightness and contrast correction appear essential. In addition, high R^2^ values for the fitting reveal appropriateness of the underlying assumption (R^2^ values of 0.9 and 0.75 for brightness and contrast fitting, respectively, for more than approximately 170 valid patches; see Supplementary Figure 18).

### Unifying the Brightness and Contrast in Superior-Inferior Direction

The constants *B*_*T*_ and *C*_*T*_ can, in principle, be chosen arbitrarily and can be used to unify the mean background intensity and contrast across slices of a three-dimensional image stack, that is, along the superior-inferior direction, which is also considered important for analysis of respective data (Gaffling et al., 2014; Yayon et al., 2018). In our experiments, we indeed did so and set the constants to identical values for all slices. It should, however, be noted that one should be careful not to over-interpret the actual intensity levels in terms of neuroscientific conclusions, for instance, by comparison of absolute intensity values across cortical layers. Similar to the explanation of the existence of potential daily variations of the vignetting effect, the amount of synapses, glia cells, vessels, etc. and, thus, the light intensity for the different slices also varies. In turn, due the similar cellular composition of slices within a specific layer compartment, it appears reasonable to compare the intensity values within a laminar compartment. For a reliable comparison across laminar compartment borders, there is, however, a lack of supporting evidence.

### Application to Calcium Imaging Data

The present approach may also be relevant for further imaging techniques such as Ca^2+^ imaging (O’Donovan et al., 1993; Svoboda et al., 1997; Wachowiak and Cohen, 2001; Stosiek et al., 2003; Birkner et al., 2017; Tischbirek et al., 2019; Yildirim et al., 2019; Chen et al., 2020). Compared to IEG-based images, which were used for illustration in this work, the application of the proposed vignetting correction protocol to calcium indicator-based imaging will face several challenges. Factors like a different signal-to-noise ratio and the spatial position of the vignetting center have theoretically little impact on the applicability of the protocol. However, an irregular spatial distribution of background noise represents a major obstacle. Unfortunately, using current calcium imaging techniques, with only a few exceptions (Chen et al., 2020), the background noise is disturbed by irregularly distributed axons and dendrites. In addition, the increased computational time required for processing large calcium imaging data sets has to be kept in mind when applying the proposed correction protocol to such data. Nonetheless, there may be potential for the current image correction approach in a variety of further structural and functional imaging techniques (Kherlopian et al., 2008; Yao and Wang, 2014).

## Data Availability Statement

The datasets presented in this study can be found in online repositories. The names of the repository/repositories and accession number(s) can be found below: https://github.com/IPMI-ICNS-UKE/VignettingCorrection.

## Ethics Statement

The animal study was reviewed and approved by Tsinghua University, Beijing, China.

## Author Contributions

J-SG and CH designed the research. DL and GW initialized the illumination correction idea. DL and RW developed the illumination correction and evaluation methodology. GW and HX worked on the animal experiments and data preparation. DL, RW, J-SG, and CH wrote the manuscript. All authors approved the manuscript.

## Conflict of Interest

The authors declare that the research was conducted in the absence of any commercial or financial relationships that could be construed as a potential conflict of interest.

## Publisher’s Note

All claims expressed in this article are solely those of the authors and do not necessarily represent those of their affiliated organizations, or those of the publisher, the editors and the reviewers. Any product that may be evaluated in this article, or claim that may be made by its manufacturer, is not guaranteed or endorsed by the publisher.
