# Supplementary material for: Single Image-Based Vignetting Correction for Improving the Consistency of Neural Activity Analysis in 2-Photon Functional Microscopy
Source: Front Neuroinform. 2022 Jan 5;15:674439. doi: 10.3389/fninf.2021.674439 (PMC8766855; doi:10.3389/fninf.2021.674439)
Supplement: Supplementary file 1 [file Data_Sheet_1.PDF]

Single Image-based Vignetting Correction for Improving the Consistency of Neural Activity Analysis in 2-Photon Functional Microscopy

Dong Li[†,*], Guangyu Wang[†], René Werner, Hong Xie, Ji-Song Guan, Claus C. Hilgetag

– Supplementary Material –

1 Supplementary Figures

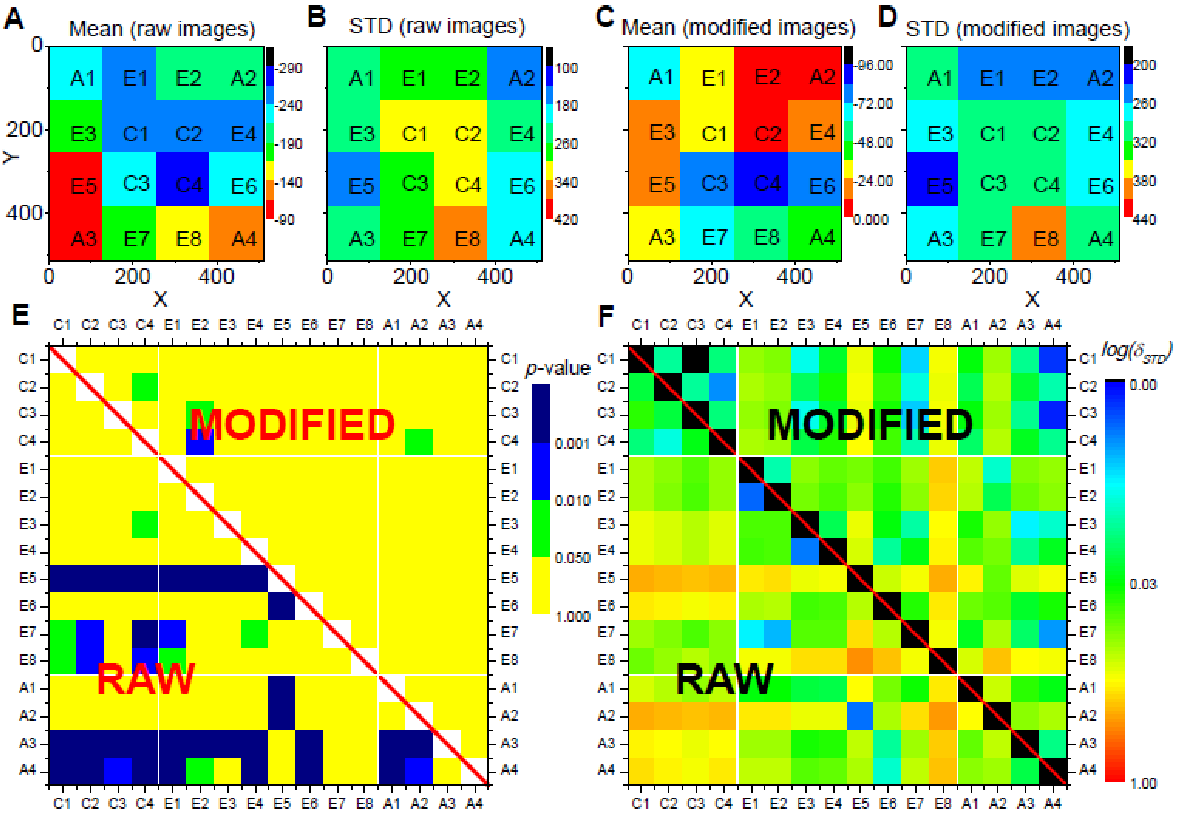

Supplementary Figure S1. Region-specific evaluation of the effects of the proposed vignetting correction on the absolute changes $\Delta X_l = X_l(day1) - X_l(day0)$ of neural activities in layers II/III of a mouse VISp area. On both days, the mouse was located in its homecage, and the interval between the measurements was 5 days. For more detailed description see the caption of Fig. 2 of the main manuscript. It can, for instance, be seen that the C-A differences in panel E in the uncorrected image ("RAW") are evaluated statistically significance (indicated by the blue color); after correction ("MODIFIED"), corresponding p-values are close to 1, illustrating that the significant differences are due to the vignetting effect.

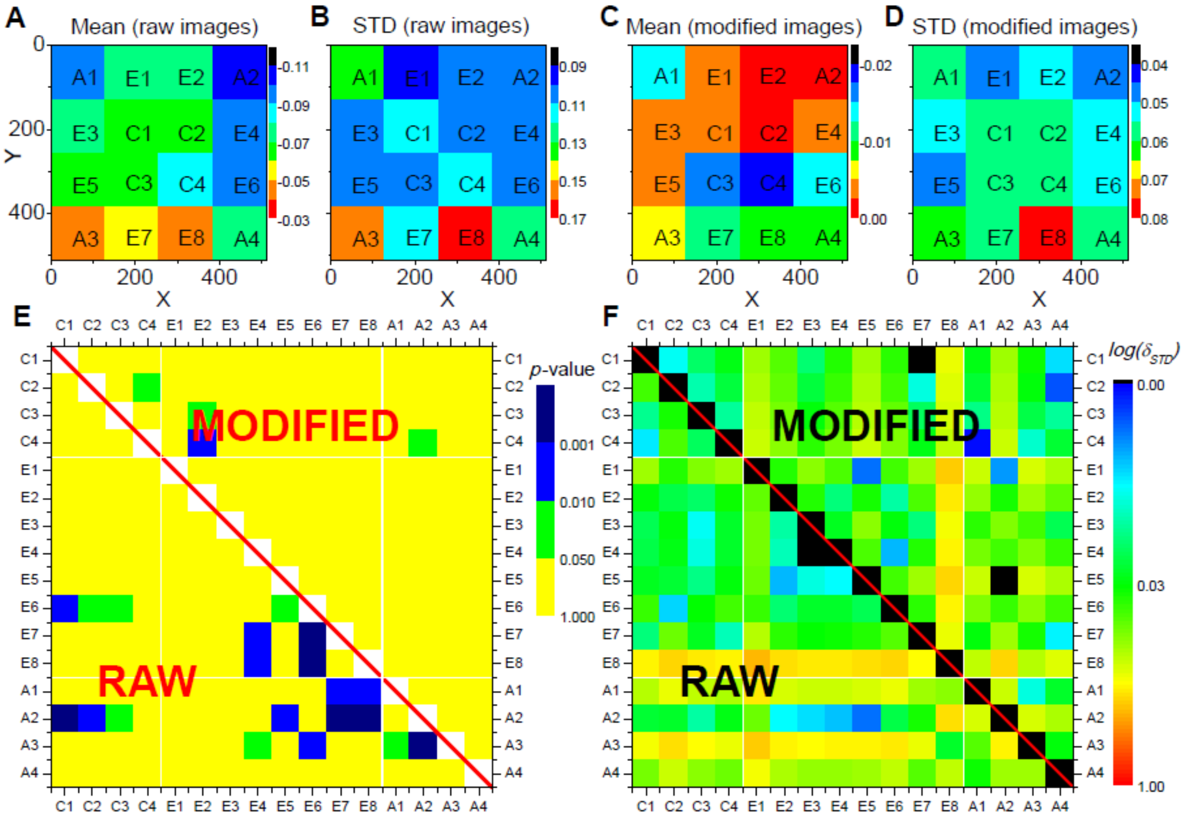

Supplementary Figure S2. Region-specific evaluation of the effects of the proposed vignetting correction on the relative changes $\boldsymbol{\delta X_l} = [\boldsymbol{X_l}(\boldsymbol{day1}) - \boldsymbol{X_l}(\boldsymbol{day0})]/[\boldsymbol{X_l}(\boldsymbol{day1}) + \boldsymbol{X_l}(\boldsymbol{day0})]$ of neural activities in layers II/III of a mouse VISp area. See Supplemental Figure S1 and corresponding caption for further explanations.

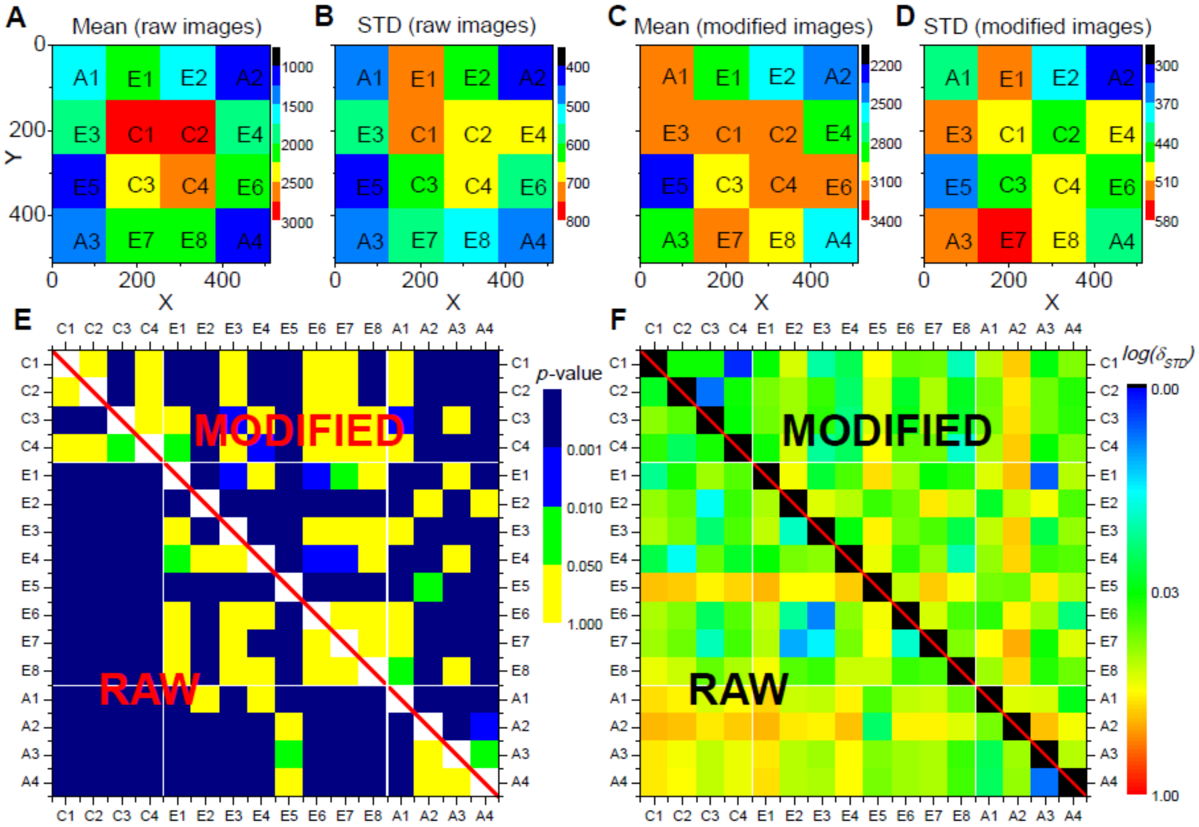

Supplementary Figure S3. Region-specific evaluation of the effects of the proposed vignetting correction on the neural activities $\boldsymbol{X_l}$ in **layer IV** of a mouse VISp area (see description of Fig. 2 for details).

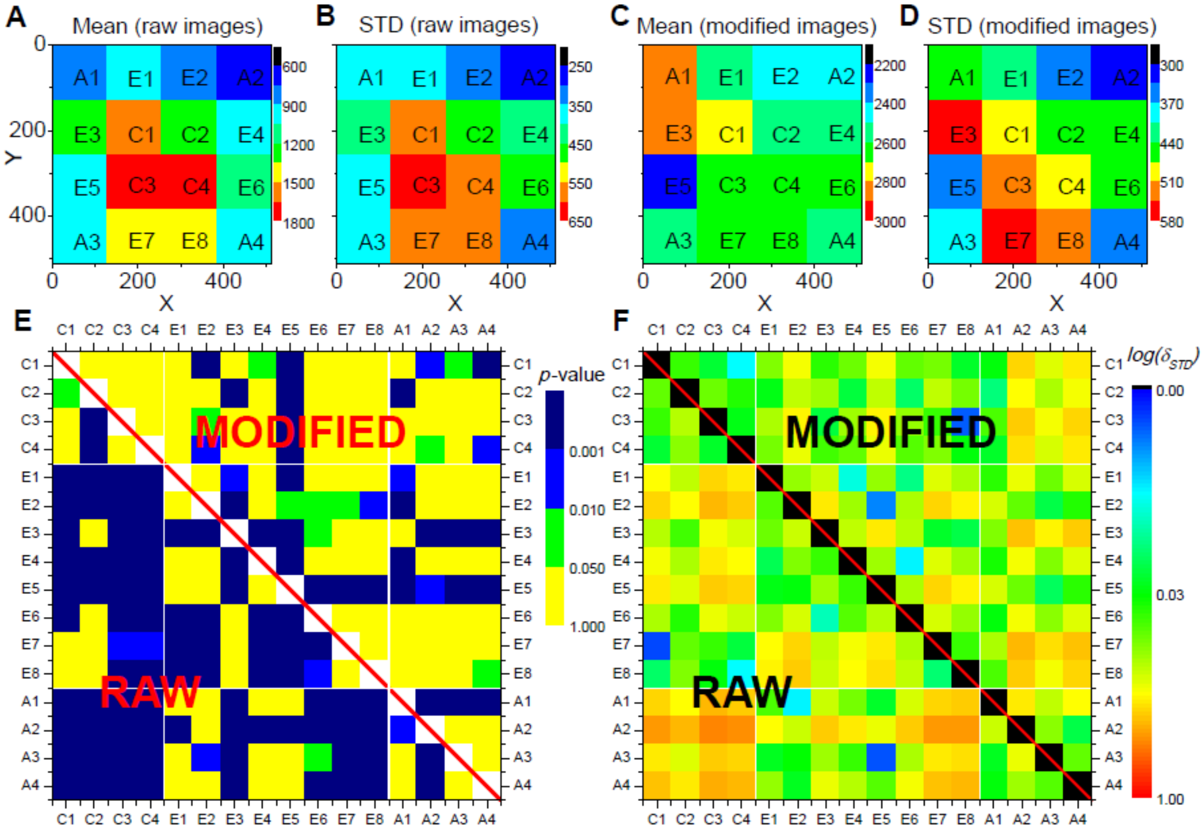

Supplementary Figure S4. Region-specific evaluation of the effects of the proposed vignetting correction on the neural activities $\boldsymbol{X_l}$ in **layer V** of a mouse VISp area.

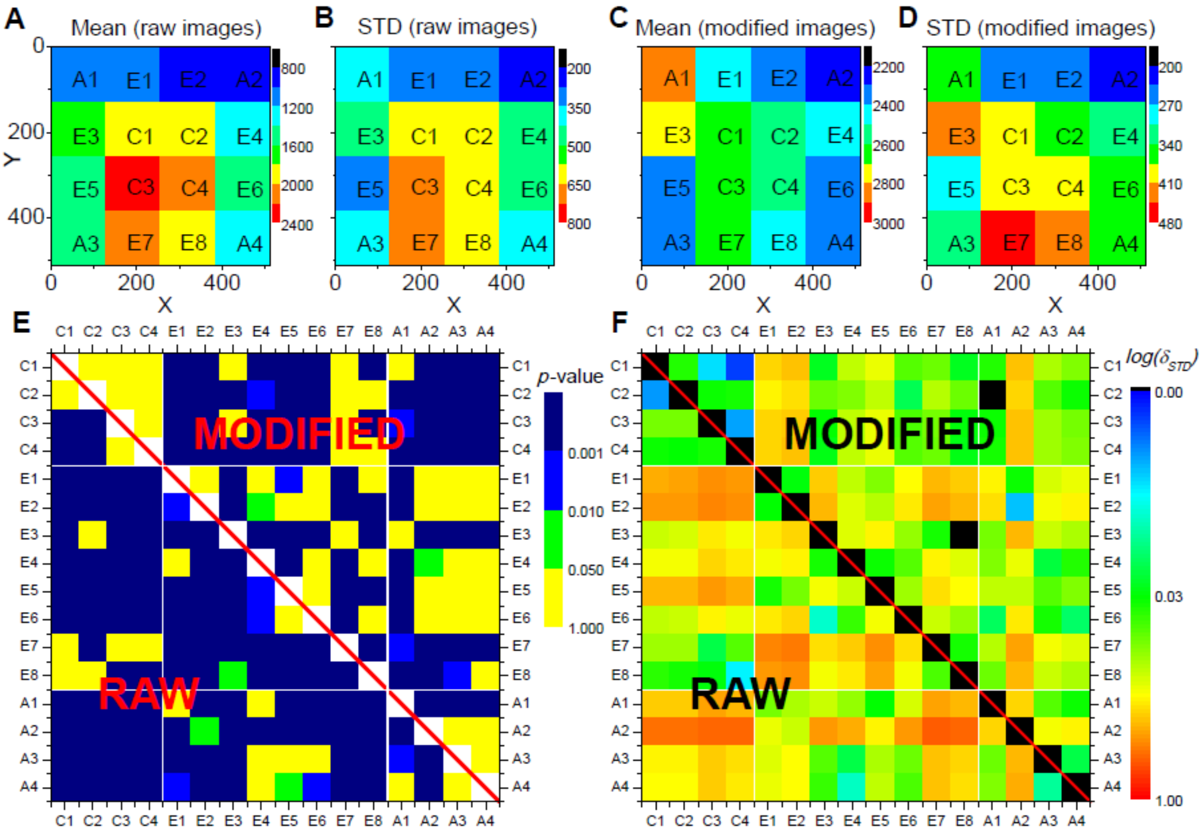

Supplementary Figure S5. Region-specific evaluation of the effects of the proposed vignetting correction on the neural activities $\boldsymbol{X_l}$ in **layer VI** of a mouse VISp area.

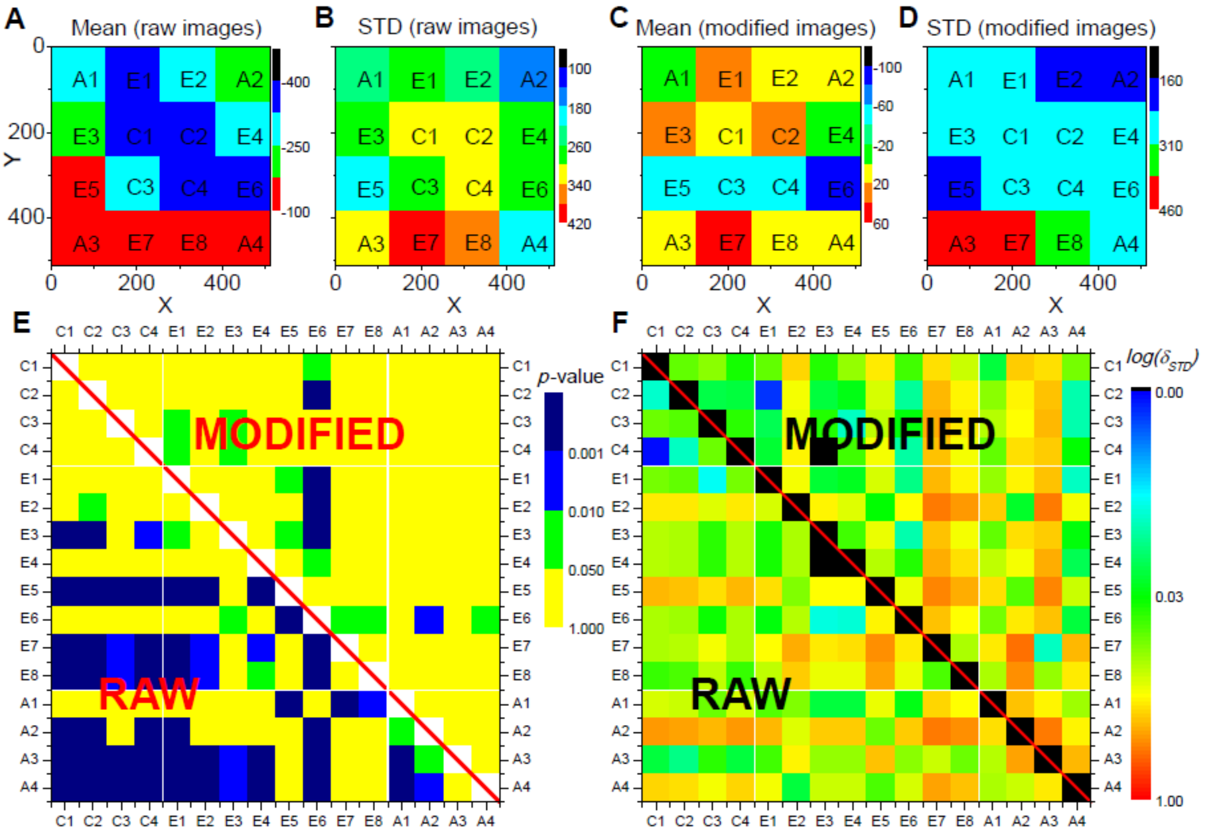

Supplementary Figure S6. Region-specific evaluation of the effects of the proposed vignetting correction on absolute changes of neural activities $\boldsymbol{\Delta X_l}$ in **layer IV** of a mouse VISp area.

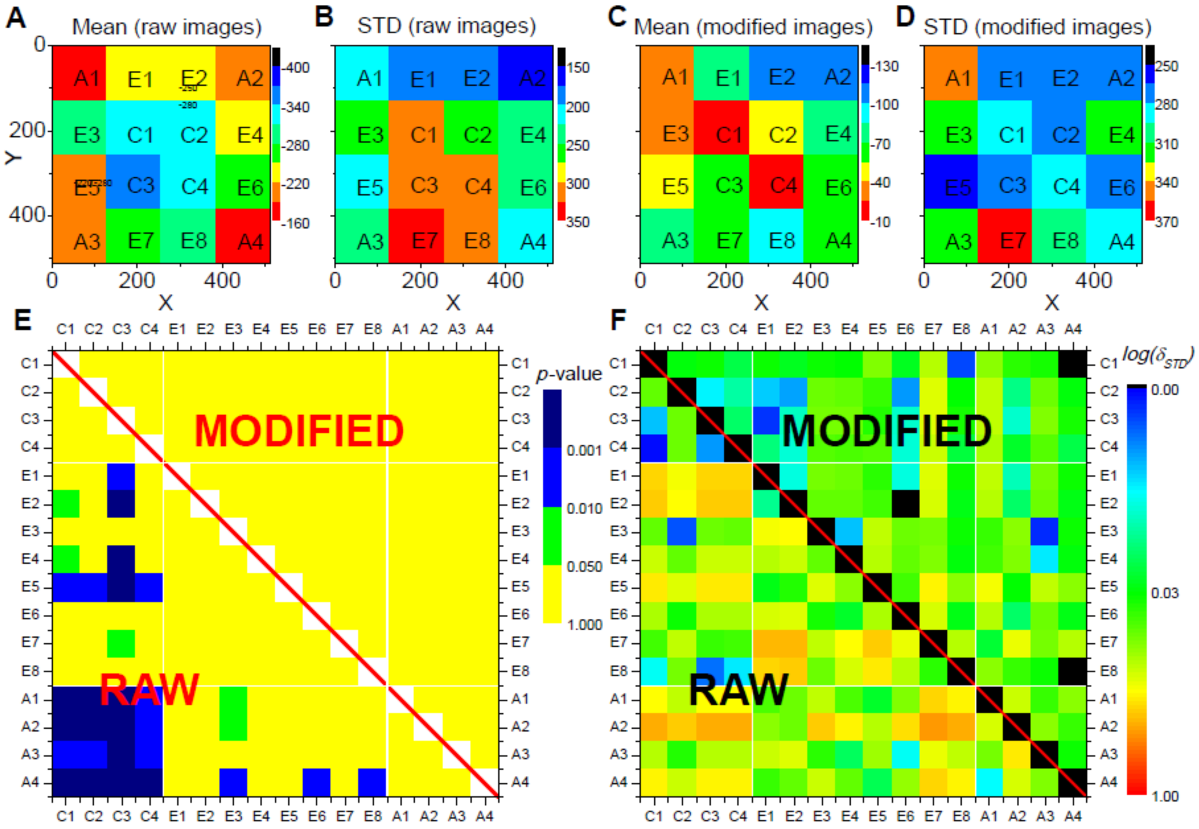

Supplementary Figure S7. Region-specific evaluation of the effects of the proposed vignetting correction on absolute changes of neural activities $\boldsymbol{\Delta X_l}$ in **layer V** of a mouse VISp area.

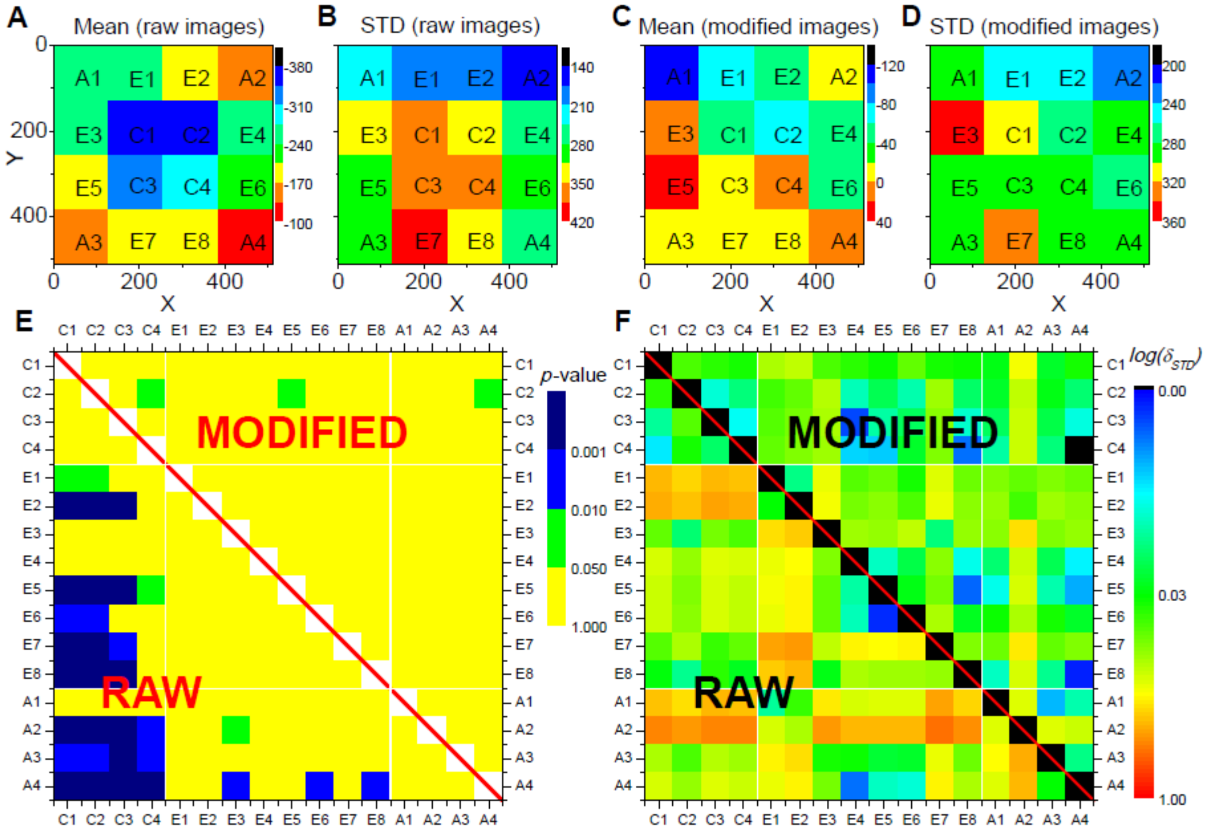

Supplementary Figure S8. Region-specific evaluation of the effects of the proposed vignetting correction on absolute changes of neural activities ΔX_l in **layer VI** of a mouse VISp area.

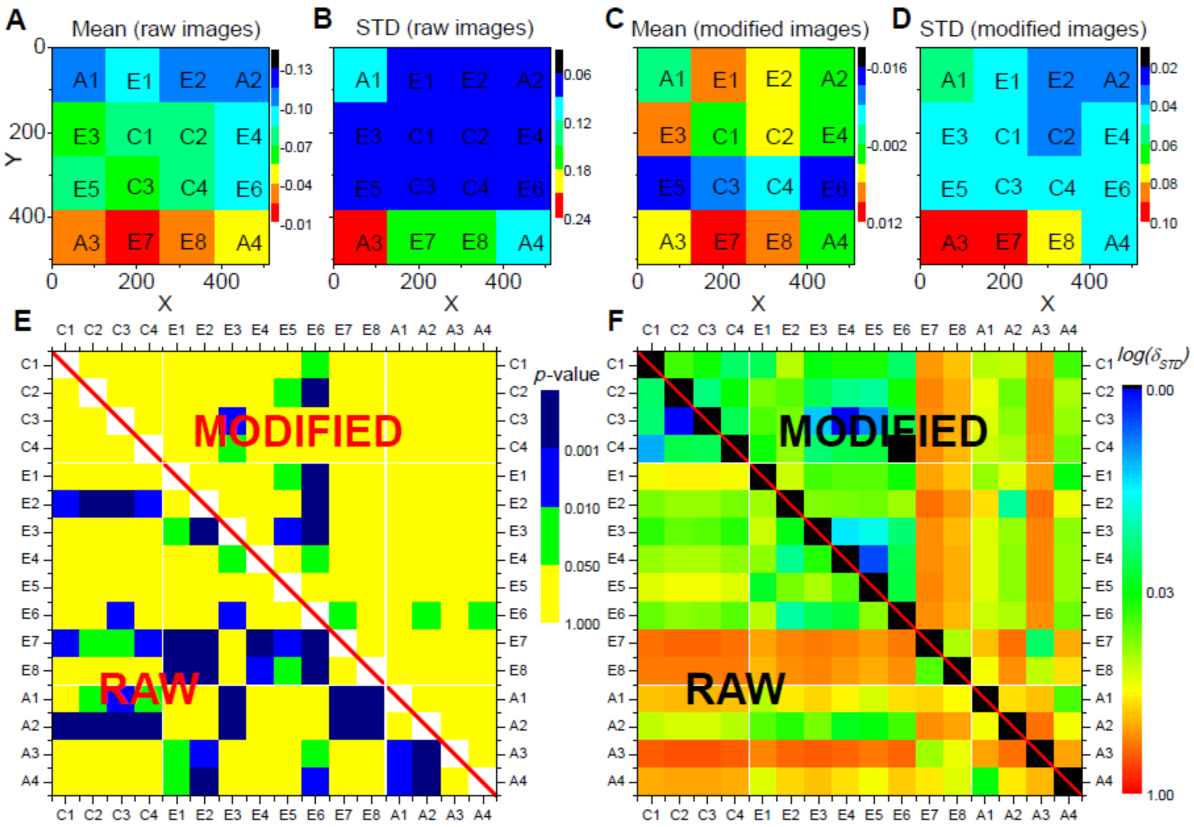

Supplementary Figure S9. Region-specific evaluation of the effects of the proposed vignetting correction on relative changes of neural activities $\boldsymbol{\delta X_l}$ in **layer IV** of a mouse VISp area.

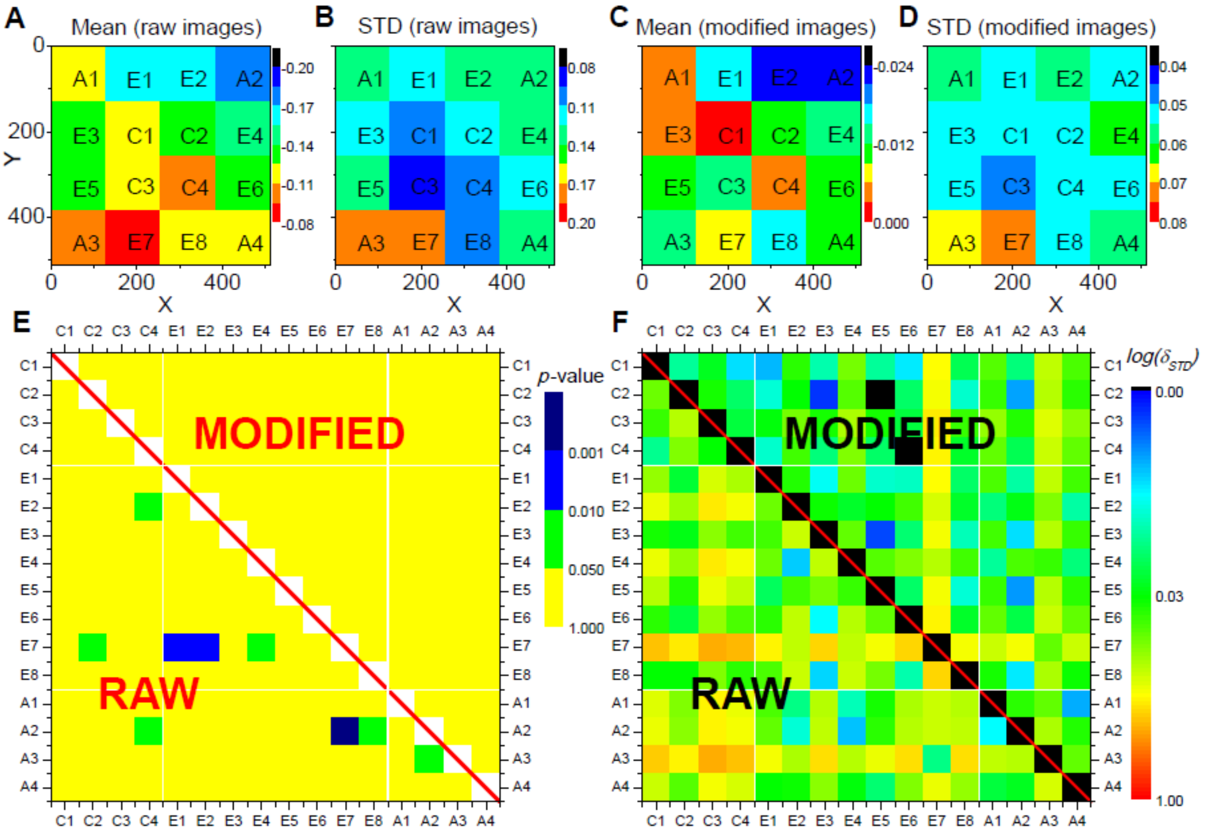

Supplementary Figure S10. Region-specific evaluation of the effects of the proposed vignetting correction on relative changes of neural activities $\boldsymbol{\delta X_l}$ in **layer V** of a mouse VISp area.

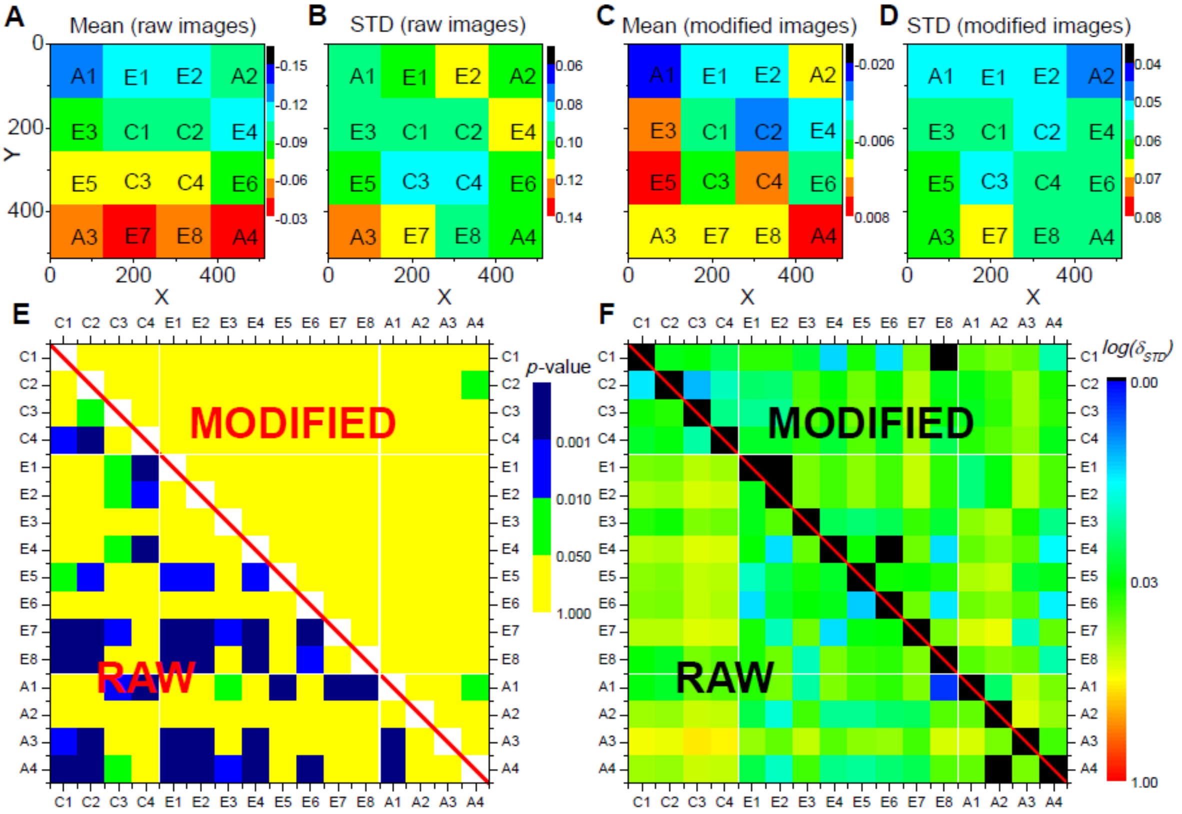

Supplementary Figure S11. Region-specific evaluation of the effects of the proposed vignetting correction on relative changes of neural activities $\boldsymbol{\delta X_l}$ in **layer VI** of a mouse VISp area.

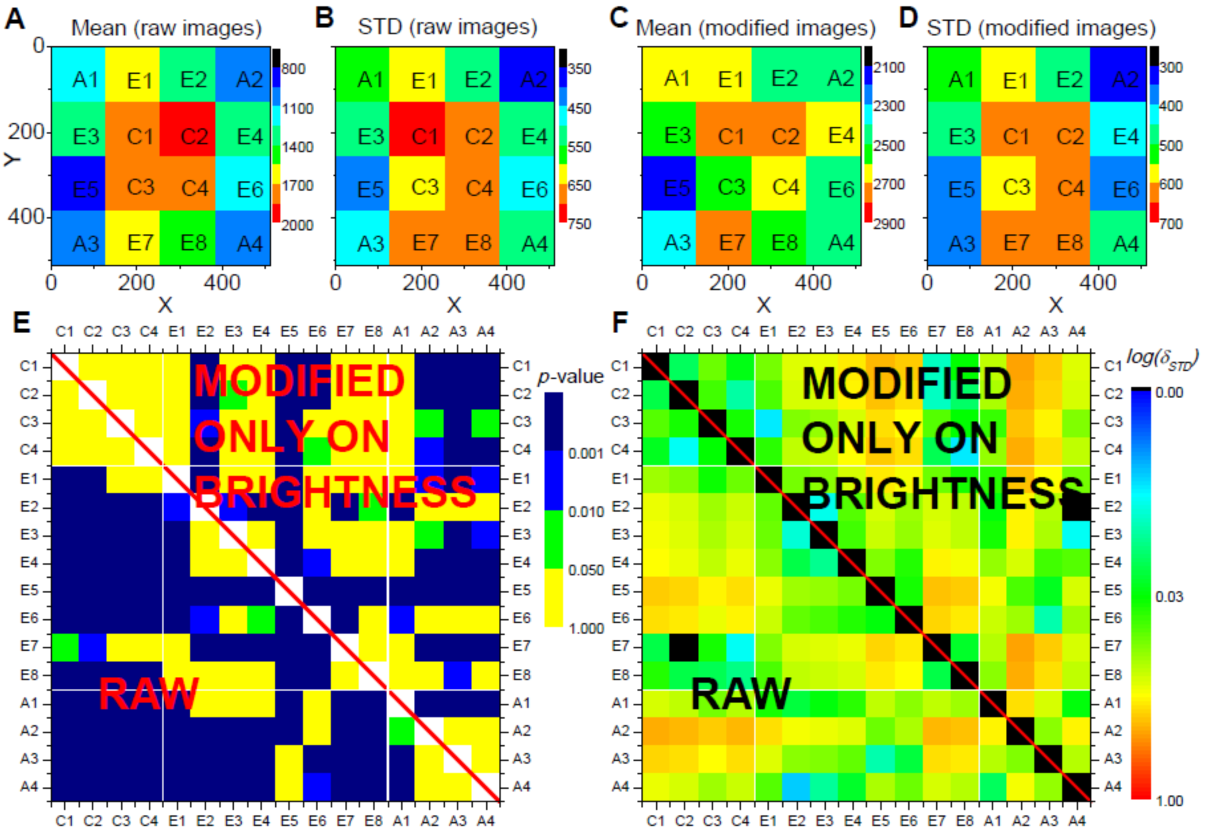

Supplementary Figure S12. Region-specific evaluation of the effects of **brightness-only correction** on neural activities $\boldsymbol{X_l}$ in **layer II/III** of a mouse VISp area.

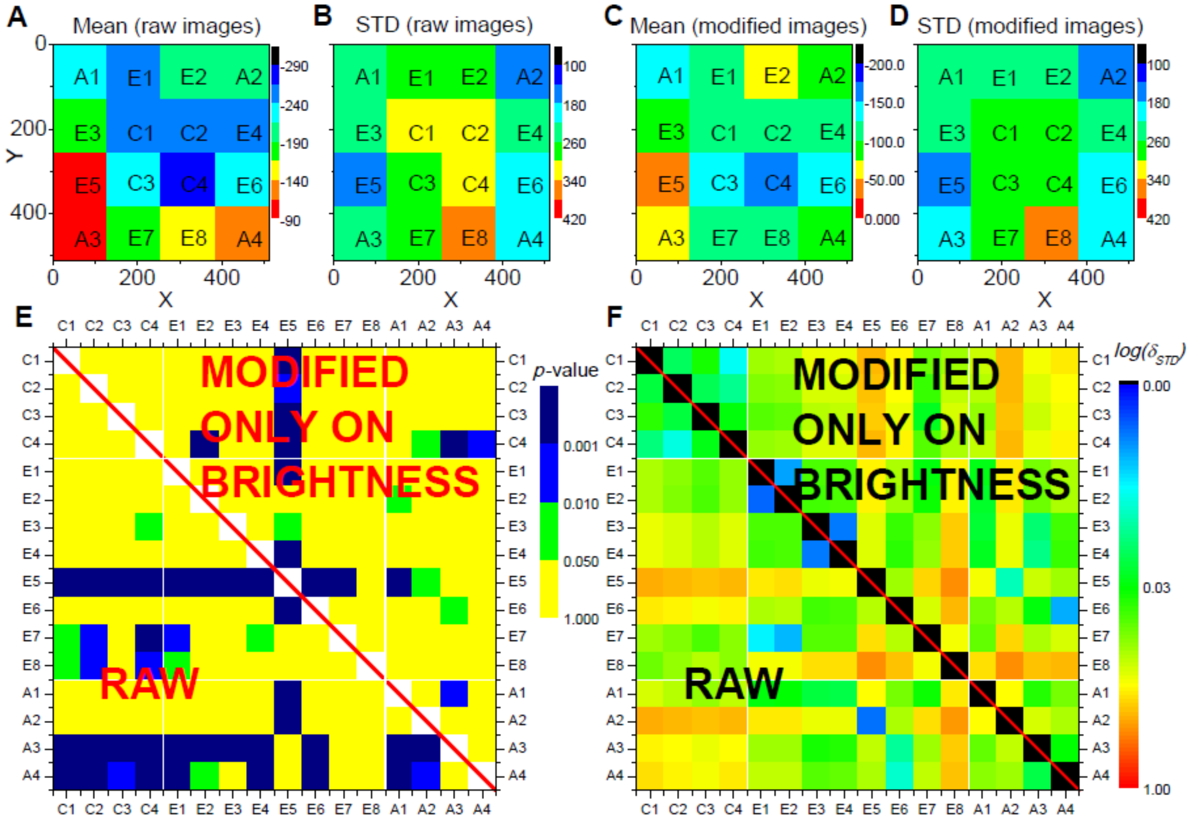

Supplementary Figure S13. Region-specific evaluation of the effects of **brightness-only correction** on absolute changes of neural activities $\boldsymbol{\Delta X_l}$ in **layer II/III** of a mouse VISp area.

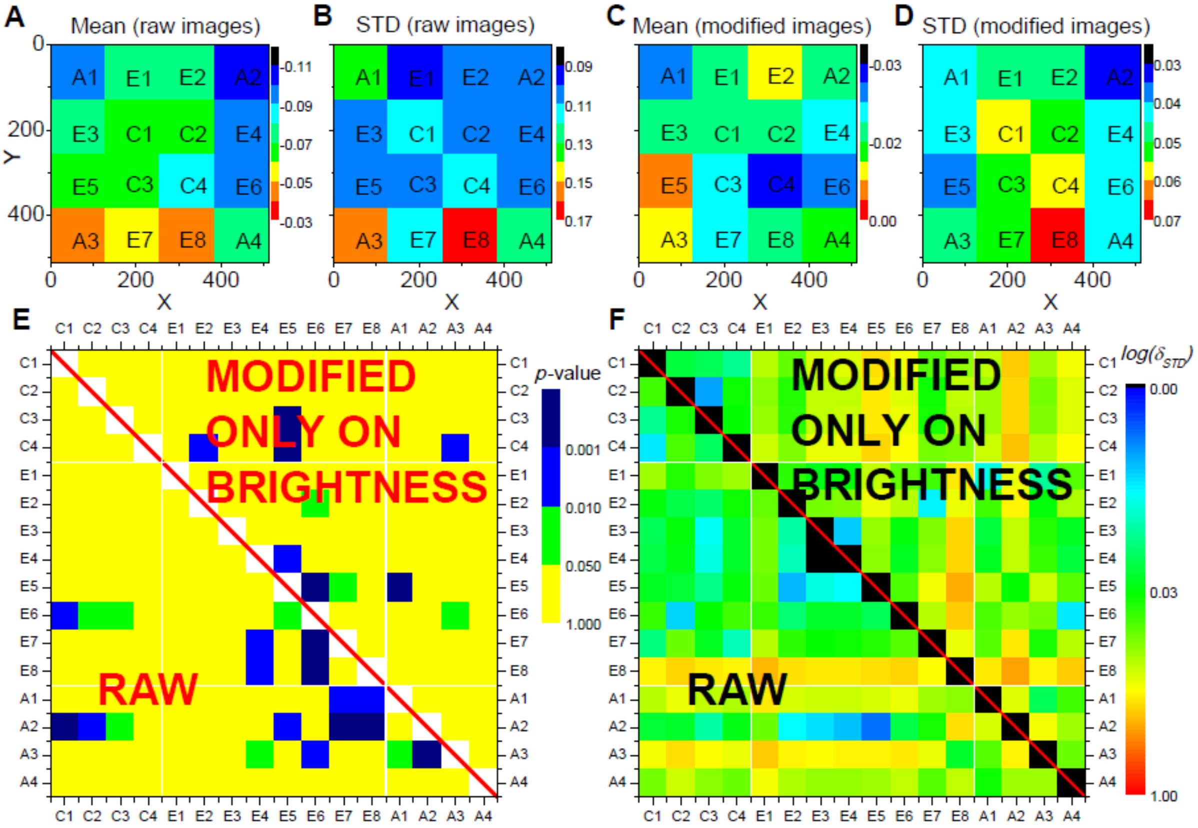

Supplementary Figure S14. Region-specific evaluation of the effects of **brightness-only correction** on relative changes of neural activities $\boldsymbol{\delta X_l}$ in **layer II/III** of a mouse VISp area.

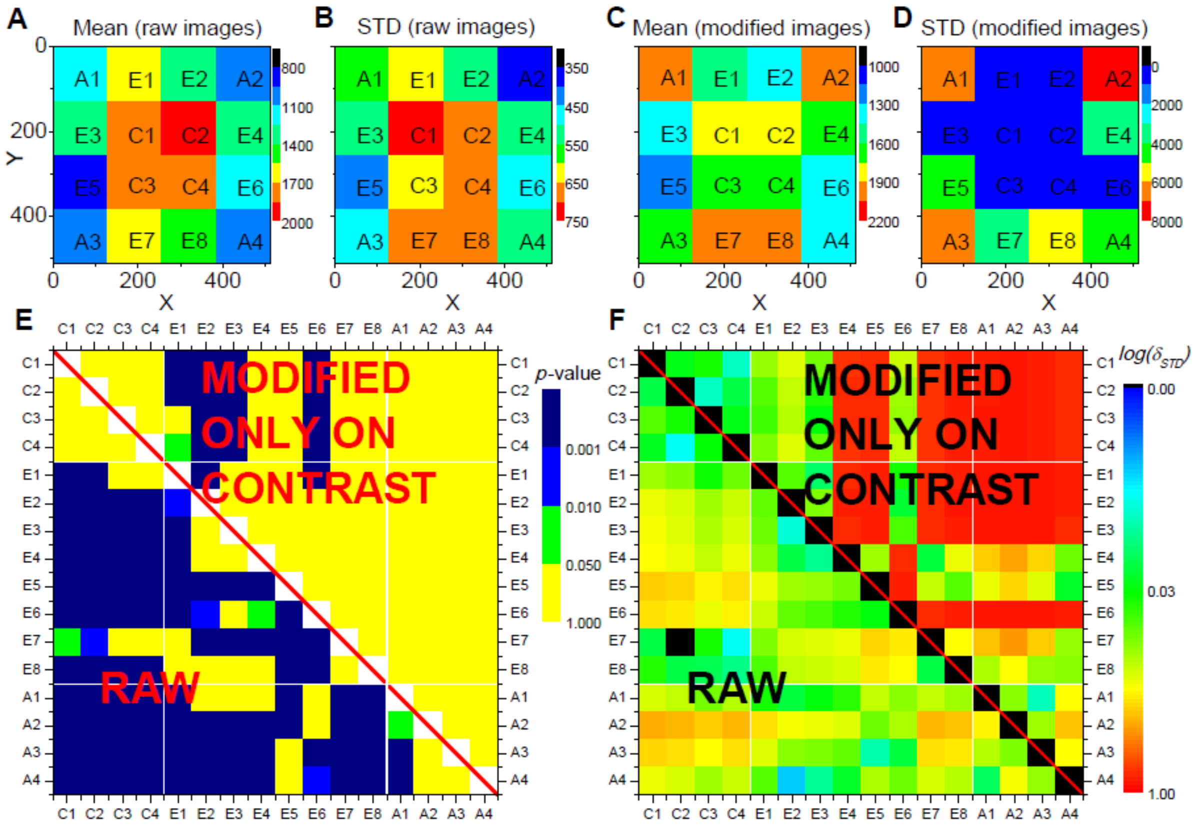

Supplementary Figure S15. Region-specific evaluation of the effects of **contrast-only correction** on neural activities $\boldsymbol{X_l}$ in **layer II/III** of a mouse VISp area.

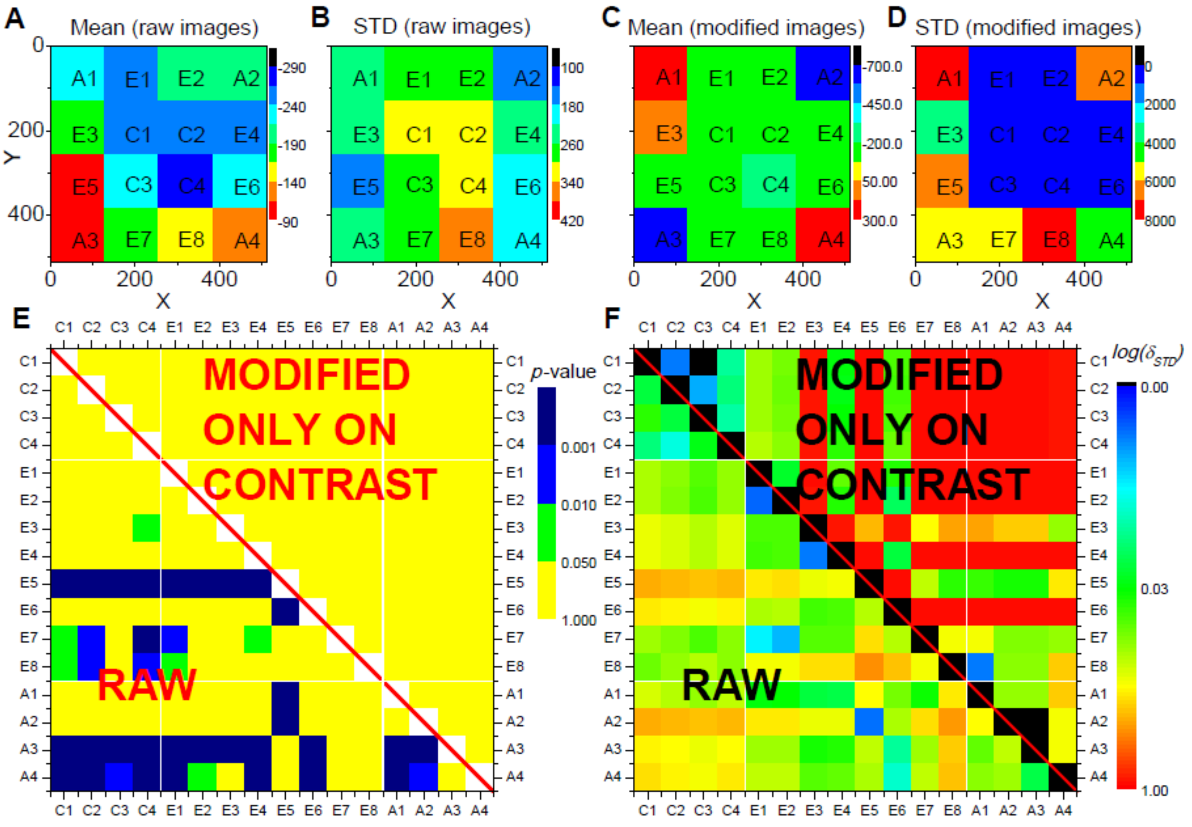

Supplementary Figure S16. Region-specific evaluation of the effects of **contrast-only correction** on absolute changes of neural activities $\boldsymbol{\Delta X_l}$ in **layer II/III** of a mouse VISp area.

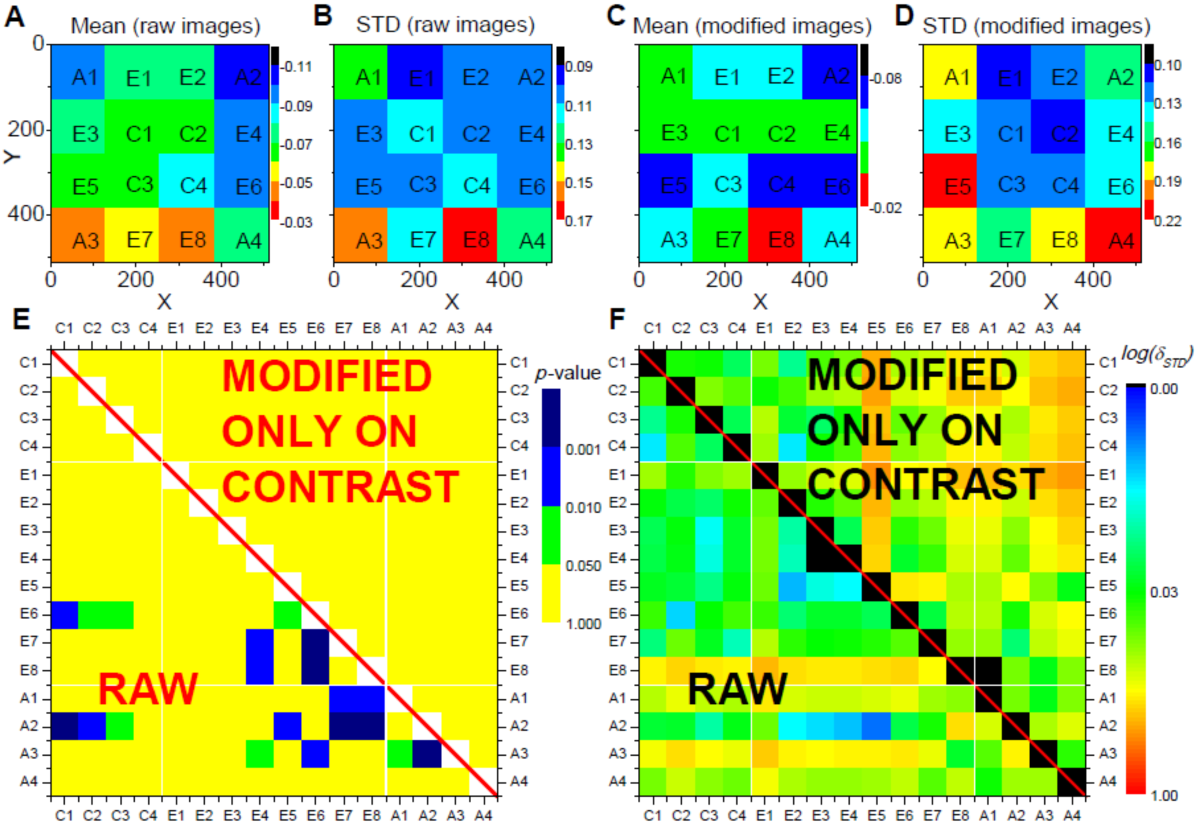

Supplementary Figure S17. Region-specific evaluation of the effects of **contrast-only correction** on relative changes of neural activities $\boldsymbol{\delta X_l}$ in **layer II/III** of a mouse VISp area.

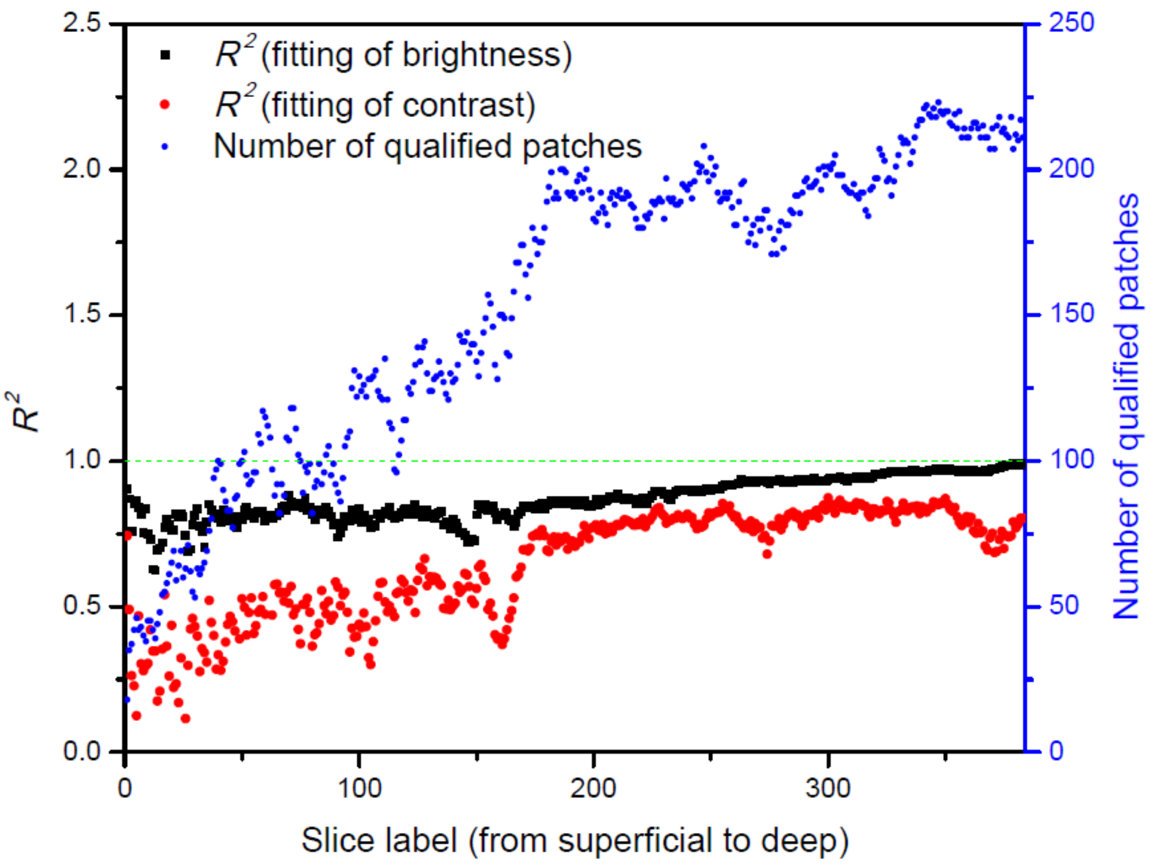

Supplementary Figure S18. $\boldsymbol{R^2}$ values of Gaussian fitting to the patch brightness values (black squares) and contrast values (red dots) in one image stack (in total 383 slices). Blue dots show the number of patches that, regarding the intensity histogram, pass the Shapiro-Wilk test ($S_{min} = 0.98$) and that are eventually used for the two-dimensional Gaussian fitting.
